# Genetic and psychosocial stressors have independent effects on the level of subclinical psychosis: findings from the multinational EU-GEI study

**DOI:** 10.1017/S2045796022000464

**Published:** 2022-09-27

**Authors:** B. Pignon, H. Peyre, A. Ayrolles, J. B. Kirkbride, S. Jamain, A. Ferchiou, J. R. Richard, G. Baudin, S. Tosato, H. Jongsma, L. de Haan, I. Tarricone, M. Bernardo, E. Velthorst, M. Braca, C. Arango, M. Arrojo, J. Bobes, C. M. Del-Ben, M. Di Forti, C. Gayer-Anderson, P. B. Jones, C. La Cascia, A. Lasalvia, P. R. Menezes, D. Quattrone, J. Sanjuán, J. P. Selten, A. Tortelli, P. M. Llorca, J. van Os, B. P. F. Rutten, R. M. Murray, C. Morgan, M. Leboyer, A. Szöke, F. Schürhoff

**Affiliations:** 1 Univ Paris Est Créteil, INSERM, IMRB, AP-HP, Hôpitaux Universitaires H. «Mondor», DMU IMPACT, Fondation FondaMental, Créteil F-94010, France; 2 AP-HP, Hôpital universitaire Robert Debré, Service de pédopsychiatrie, Paris 75019, France; 3 PsyLife Group, Division of Psychiatry, UCL, London W1T 7NF, UK; 4 Université de Paris, Laboratoire de Psychopathologie et Processus de Santé, Boulogne Billancourt F-92100, France; 5 Section of Psychiatry, Department of Neuroscience, Biomedicine and Movement Sciences, University of Verona, Verona, Italy; 6 Centre for Transcultural Psychiatry ‘Veldzicht’, Balkbrug, the Netherlands; 7 VR Mental Health Group, University Center for Psychiatry, Univerisity Medical Centre Groningen, Groningen, the Netherlands; 8 Centre for Longitudinal Studies, UCL, London, UK; 9 Amsterdam UMC, Amsterdam, The Netherlands; 10 Arkin, Amsterdam, The Netherlands; 11 Department of Medical and Surgical Sciences, Bologna University, Bologna, Italy; 12 Barcelona Clínic Schizophrenia Unit, Hospital Clínic of Barcelona, Institute of Neuroscience, University of Barcelona, Barcelona, Spain; 13 Institut d'investigacions Biomèdiques August Pi i Sunyer (IDIBAPS), Barcelona, Spain; 14 Centro de Investigación Biomédica en Red de Salud Mental (CIBERSAM), Madrid, Spain; 15 Department of Psychiatry, Icahn School of Medicine at Mount Sinai, New York, USA; 16 Department of Mental Health and Pathological Addictions, Local Health Authority, Bologna 40100, Italy; 17 Department of Child and Adolescent Psychiatry, Institute of Psychiatry and Mental Health, Hospital General.Universitario Gregorio Marañón, Gregorio Marañón, (IiGSM), School of Medicine, Universidad Complutense de Madrid, Madrid, Spain; 18 CIBERSAM, Madrid, Spain; 19 Department of Psychiatry, Psychiatric Genetic Group, Instituto de Investigación Sanitaria de Santiago de Compostela, Complejo Hospitalario Universitario de Santiago de Compostela, Santiago de Compostela, Spain; 20 Faculty of Medicine and Health Sciences – Psychiatry, Universidad de Oviedo, Oviedo, Spain; 21 ISPA, INEUROPA CIBERSAM, Oviedo, Spain; 22 Department of Neuroscience and Behaviour, Ribeirão Preto Medical School, University of São Paulo, São Paulo, Brazil; 23 Social, Genetic and Developmental Psychiatry Centre, Institute of Psychiatry, Psychology and Neuroscience, King's College London, London SE5 8AF, UK; 24 South London and Maudsley NHS Mental Health Foundation Trust, London, UK; 25 Department of Health Service and Population Research, Institute of Psychiatry, King's College London, De Crespigny Park, Denmark Hill, London SE5 8AF, UK; 26 Department of Psychiatry, University of Cambridge, Cambridge CB2 0SZ, UK; 27 CAMEO, Cambridgeshire & Peterborough NHS Foundation Trust, Cambridge, UK; 28 Department Biomedicine, Neuroscience and Advanced Diagnostics, School of Medicine, University of Palermo, Palermo, Italy; 29 Department of Preventive Medicine, Faculdade de Medicina, Universidade of São Paulo, São Paulo, Brazil; 30 Social, Genetic, and Developmental Psychiatry Centre, Institute of Psychiatry, Psychology and Neuroscience, King's College London, London, UK; 31 Biomedical Research Networking Centre in Mental Health (CIBERSAM), Madrid, Spain; 32 Department of Psychiatry, Hospital Clínico Universitario de Valencia, School of Medicine, Universidad de Valencia, Valencia, Spain; 33 Biomedical Research Institute INCLIVA, Valencia, Spain; 34 Rivierduinen Institute for mental Health, Leiden, The Netherlands; 35 Maastricht University Medical Center, Department of Psychiatry & Neuropsychology, School for Mental Health and Neuroscience, Maastricht, The Netherlands; 36 French National Institute of Health and Medical Research (INSERM), U955 – 15, Créteil, France and EPS Maison Blanche, Paris, France; 37 CHU Clermont-Ferrand, Psychiatrie B, Clermont-Ferrand F-63003, France; 38 Université Clermont Auvergne, EA7280, Clermont-Ferrand F-63000, France; 39 Department of Psychiatry and Neuropsychology, School for Mental Health and Neuroscience, Maastricht University Medical Centre, Maastricht, The Netherlands; 40 Department of Psychiatry, UMC Utrecht Brain Centre, University Medical Centre Utrecht, Utrecht University, Utrecht, The Netherlands; 41 Department of Psychosis Studies, Institute of Psychiatry, Psychology & Neuroscience, King's College London, London, UK; 42 The Institute of Psychiatry, Psychology and Neuroscience, King's College London, UK

**Keywords:** Genes × environment interactions, polygenic risk score for schizophrenia, psychosis, psychosocial stressors

## Abstract

**Aims:**

Gene x environment (G×E) interactions, i.e. genetic modulation of the sensitivity to environmental factors and/or environmental control of the gene expression, have not been reliably established regarding aetiology of psychotic disorders. Moreover, recent studies have shown associations between the polygenic risk scores for schizophrenia (PRS-SZ) and some risk factors of psychotic disorders, challenging the traditional gene *v.* environment dichotomy. In the present article, we studied the role of GxE interaction between psychosocial stressors (childhood trauma, stressful life-events, self-reported discrimination experiences and low social capital) and the PRS-SZ on subclinical psychosis in a population-based sample.

**Methods:**

Data were drawn from the EUropean network of national schizophrenia networks studying Gene-Environment Interactions (EU-GEI) study, in which subjects without psychotic disorders were included in six countries. The sample was restricted to European descendant subjects (*n* = 706). Subclinical dimensions of psychosis (positive, negative, and depressive) were measured by the Community Assessment of Psychic Experiences (CAPE) scale. Associations between the PRS-SZ and the psychosocial stressors were tested. For each dimension, the interactions between genes and environment were assessed using linear models and comparing explained variances of ‘Genetic’ models (solely fitted with PRS-SZ), ‘Environmental’ models (solely fitted with each environmental stressor), ‘Independent’ models (with PRS-SZ and each environmental factor), and ‘Interaction’ models (Independent models plus an interaction term between the PRS-SZ and each environmental factor). Likelihood ration tests (LRT) compared the fit of the different models.

**Results:**

There were no genes-environment associations. PRS-SZ was associated with positive dimensions (*β* = 0.092, *R*^2^ = 7.50%), and most psychosocial stressors were associated with all three subclinical psychotic dimensions (except social capital and positive dimension). Concerning the positive dimension, Independent models fitted better than Environmental and Genetic models. No significant GxE interaction was observed for any dimension.

**Conclusions:**

This study in subjects without psychotic disorders suggests that (i) the aetiological continuum hypothesis could concern particularly the positive dimension of subclinical psychosis, (ii) genetic and environmental factors have independent effects on the level of this positive dimension, (iii) and that interactions between genetic and individual environmental factors could not be identified in this sample.

## Introduction

Both environmental and genetic factors are associated with an increased risk of developing psychotic disorders (van Os *et al*., 2010). The relationships between these factors have long been discussed, and the hypothesis of genes x environment (GxE) interactions was suggested several decades ago (Strahilevitz, 1974; Murray *et al*., 1986; Schulsinger *et al*., 1987). Such interaction can be defined as a genetic modulation of the sensitivity to environmental factors and/or environmental control of the gene expression (Kendler and Eaves, 1986). Numerous studies supported this hypothesis (Collip *et al*., 2013; Frydecka *et al*., 2020; Pries *et al*., 2020*a*), and particularly one from Caspi *et al*. (2005), in which a significant interaction between cannabis use in adolescence and the genetic variant Val*^158^*Met in the Catechol-O-Methyltransferase (COMT, which metabolises dopamine) gene was found. In this study, in comparison to Val/Val genotype, Met/Met and Met/Val genotypes had a protective effect against the risk of psychotic symptoms and disorders among cannabis users (in the group of subjects without cannabis use, the rates of psychotic symptoms and disorders were similar according to the different genotypes). Of note, although discrepant results have been reported in replication studies (Henquet *et al*., 2006; Zammit *et al*., 2007), a meta-analysis confirmed the small protective effect of the Val/Met heterozygous genotype [pooled OR = 0.947 95% CI (0.904–0.993), *p* = 0.023] (Costas *et al*., 2011). This meta-analysis, that did not take account of the cannabis use, hypothesised that both too high and too low levels of dopamine could be risk factors. Study of GxE interactions is difficult due to the need for large cohorts with well-characterised genetic and environmental data.

To deal with these difficulties, the study of subclinical psychosis in the general population, that can be defined as psychotic symptoms in subjects who do not meet criteria for psychotic disorders, is convenient (Verdoux and van Os, 2002; McGrath *et al*., 2015), especially in accordance to the aetiological psychotic continuum hypothesis. According to this hypothesis, subclinical psychosis has a similar origin/aetiology as psychotic disorders (Linscott and van Os, 2013; van Os, 2014; Pignon *et al*., 2018; Pries *et al*., 2018). Thus, studying genetic or environmental risk factors associated with subclinical psychosis may provide insights into the aetiology of psychosis and partly reduce the potential interference of reverse causation, i.e. factors are associated with or caused by the clinical disorders themselves [e.g. hospitalisations, stigma, substance use disorders or social drift after onset (Zipursky, 2014; Sariaslan *et al*., 2016; Pignon *et al*., 2019*a*)]. Furthermore, in line with the continuum theory, subclinical psychosis can be characterised by continuous variables, improving statistical power, which is a key issue in GxE interaction studies.

Psychotic disorders are characterised by a polygenic architecture, with thousands of common genetic variants with small effect sizes, and a few rare variants with large effect sizes (Smeland *et al*., 2020). The genome-wide effects of disease-associated common genetic variants can be summarised in a polygenic risk score (PRS) (Anderson *et al*., 2019), which offers new opportunities to characterise the complex genetic aetiology of psychotic disorders. In subjects included through the EUropean network of national schizophrenia networks studying Gene-Environment Interactions (EU-GEI), the PRS for schizophrenia (PRS-SZ) explained between 7 and 9% of the variance of the case-control status (Di Forti *et al*., 2019*b*; Tripoli *et al*., 2020), consistently with other studies (Vassos *et al*., 2017). Of note, among patients with psychotic disorders, the PRS-SZ is also associated with antipsychotic treatment response, the level of quality of life, or, in the general population, to the intelligence quotient (IQ), and the risk of attention-deficit/hyperactivity disorder (ADHD) (Mistry *et al*., 2018; Legge *et al*., 2019; Zhang *et al*., 2019; Pries *et al*., 2020*c*).

Studies of associations between subclinical psychosis and the PRS-SZ have produced contradictory results (Zammit *et al*., 2014; Mistry *et al*., 2018; Legge *et al*., 2019; Nenadić *et al*., 2020), and further studies are needed. Moreover, to date, four studies have investigated the role of GxE interaction on subclinical psychosis using PRS-SZ. Two studies assessed the interaction between PRS-SZ and childhood trauma, but only one reported a significant interaction (Pries *et al*., 2020*b*), whereas the other showed an independent (additive) effects of the PRS-SZ and the trauma without significant interaction (Trotta *et al*., 2016). A recent study assessing the associations between momentary stress and subclinical psychotic symptoms showed that higher levels of PRS-SZ were associated with a higher intensity of symptoms after a momentary stress among controls (Schick *et al*., 2022). In the fourth study, the authors assessed the interaction between PRS-SZ and smoking status, but did not show significant association (García-González *et al*., 2020).

In addition to increasing the risk for psychosis by GxE interactions, the PRS-SZ has also been associated with a greater risk of exposure to environmental risk factors for psychosis (Pingault *et al*., 2018). For instance, several studies have reported associations between the PRS-SZ and cannabis use (Gage *et al*., 2017; Pasman *et al*., 2018) or between the PRS-SZ and urbanicity (Colodro-Conde *et al*., 2018; Paksarian *et al*., 2018; Maxwell *et al*., 2021) or the level of neighbourhood deprivation and social fragmentation at birth (Solmi *et al*., 2020), challenging the traditional gene *v.* environment dichotomy. However, these observations could not explain the strength of the associations between cannabis use or urbanicity and the risk of psychotic disorders (Vassos *et al*., 2012; Di Forti *et al*., 2019*a*). To the best of our knowledge, the genetic-environment (G-E) associations between PRS-SZ and psychosocial stressors have not been studied to date.

In a former study on population-based controls from the EU-GEI work package 2 (WP2) (Pignon *et al*., 2021), we showed that psychosocial stressors, i.e. childhood trauma, stressful life-events, self-reported discrimination experiences and low social capital, had independent effects on subclinical psychosis dimensions, without significant environment x environment (ExE) interactions. In the current study, we aimed to study the relationships between these psychosocial stressors, the PRS-SZ, and three dimensions of subclinical psychosis (positive, negative, depressive), looking for GxE interaction. Furthermore, we aimed to study the association between psychosocial stressors and the PRS-SZ, looking for G-E associations.

## Methods

### EU-GEI WP2 study

Clinical, environmental and genetic data have been collected through the EU-GEI WP2 (named ‘*Functional Enviromics*’), a multicentre case-sibling-control study of genetic and environmental determinants of the occurrence and severity of psychotic disorders. Population-based controls were recruited across 6 countries: Brazil, France, Italy, the Netherlands, Spain, and the United Kingdom. Inclusion criteria were: age 18–64, living in the catchment areas, no evidence of current or past psychotic disorders. These controls were recruited using a mixture of random and quota sampling to ensure that they were broadly representative of the at-risk populations on predefined variables (age, sex, and migration) (Gayer-Anderson *et al*., 2020).

Ethical approval was obtained from local research ethics committees in each country. The EU-GEI Project was funded by the European Community's Seventh Framework Program under grant agreement no. *HEALTH-F2-2010-241909*.

### Subclinical psychosis and psychosocial stressors assessment

The Community Assessment of Psychic Experiences (CAPE) is a 42-item self-report questionnaire that has been developed to assess lifetime subclinical psychotic dimensions in the general population (Stefanis *et al*., 2002). For each item, 4 answers were possible according to the frequency of their occurrences (from never to nearly always). To construct the dimension scores [positive, negative and depressive (Mark and Toulopoulou, 2016)], we dichotomised answers of each CAPE item (never *v.* sometimes or more) and summed the positive answers. The cross-national invariance of the CAPE score in the EU-GEI WP2 samples was previously demonstrated (Pignon *et al*., 2019*b*).

Childhood trauma was assessed with a short version of the Childhood Trauma Questionnaire (CTQ), with 25 items assessing five different domains (emotional and physical neglect, emotional, physical and sexual abuse) (Bernstein *et al*., 2003). Only the total score was used. Lifetime self-reported discrimination experiences were assessed with a modified version of the 12-item Williams' major experiences of discrimination scale (unfairly fired or not hired because of your ethnicity/sex/weight/etc., unfairly stopped/questioned/physically threatened or abused by the police, etc.) (Williams *et al*., 1997; Jongsma *et al*., 2020). Perceived social capital in each participant's immediate neighbourhood was assessed using the Social Environment Assessment Tool (SEAT), a 23-item questionnaire, that was designed to capture four dimensions of social capital: civic disorder (CD), impact of civic disorder (ICD), informal social control (ISC), and social cohesion and trust (SCT) (Sampson *et al*., 1997; Lochner *et al*., 1999; McCulloch, 2003; Drukker *et al*., 2006). Subjects answer according to a five-point Likert-scale (1: unusual, to 5: very common), and a sum of the weighted scores of the 4 subscales were calculated to obtain the total social capital score (SEAT score = zCD + 0.51 × zICD + 1.6 × zISC + zSCT). Finally, stressful life events were assessed using the List of Threatening Experiences (LTE) which comprises 20 binary items of events usually associated with major stress over the course of the previous 6 months (e.g. serious injury, death of a parent, separation from a partner, financial difficulties) (Brugha *et al*., 1985; Motrico *et al*., 2013).

### Calculation of a polygenic risk score for schizophrenia (PRS-SZ)

Blood samples of the control sample were genotyped by the Medical Research Council Centre for Neuropsychiatric Genetics and Genomics (Cardiff, United Kingdom) using a custom ‘*Illumina HumanCoreExome-24 BeadChip*’ genotyping array, covering 570 038 genetic variants. As described elsewhere (Di Forti *et al*., 2019*b*), the PRS-SZ were generated using PRSice from the summary results of the Psychiatric Genomics Consortium (PGC), wave 2 (Schizophrenia Working Group of the PGC, 2014). Clumping was performed to obtain SNPs in approximate linkage disequilibrium with an *r*^2^ < 0.25 within a 250 kb window. PRS-SZ were calculated, at *p*-value thresholds of 0.05, because this threshold best captures liability to the disorder according to the PGC analysis (Schizophrenia Working Group of the PGC, 2014). The sample was restricted to 706 European descendant subjects (due to over-representation of European descendant subjects in the PGC2 training sample used to calculate the PRS-SZ).

### Statistical analyses

The G-E association has been assessed by Spearman correlation tests between the 4 psychosocial stressors and the PRS-SZ. Then, linear regression models were used to assess the relationships between the CAPE dimensions scores (positive, negative, depressive), environmental and genetic variables, and to look for GxE interactions. Of note, we consider multiplicative interactions (Rothman *et al*., 1980; VanderWeele and Knol, 2014).

The different models were adjusted for age, sex, and the 10 first principal components (PCs) of the genetic analyses of the ethnic variance. For each CAPE dimension, thirteen models were tested:

1. A ‘*Genetic model*’, with the sole PRS-SZ;
2. Four ‘*Environmental models*’ for each of the 4 psychosocial stressors variables: childhood trauma, stressful life-events, self-reported discrimination experiences and low social capital;
3. Four ‘*Independent models*’: one for each of the 4 psychosocial stressors variables and the PRS-SZ, without interaction term;
4. Four ‘*Interaction models*’: each of the 4 psychosocial stressors variables and the PRS-SZ, with a GxE interaction term.

To compare the fit of the different models (and particularly the Independent and the Interaction models), we compared the explained variances (*R*^2^), and use likelihood ration test (LRT) to assess whether the addition of a factor (E + G *v.* G, E + G *v.* E, E + G + E × G *v.* E + G) improved the fit of the model. To verify that the results were not biased by the imputation of the outcome (CAPE scales), analyses were repeated in a sample without imputation of the CAPE.

Psychosocial variables and PRS-SZ were standardised to Z-scores (i.e. to a mean equal to 0, and a standard-deviation equal to 1). The SEAT (social capital) score was inverted, so that higher scores were associated with *lower* social capital. Missing data of the CAPE (between 3 and 5.9% according to the different dimensions) and the psychosocial stressors variables (between 0.5 and 20.7%) were imputed with multivariate imputation by chained equations (MICE) in 20 resamples (the country was added to CAPE and psychosocial stressors variables for the imputation). R software version 3.6.0 was used for the statistical analyses.

## Results

### Description of the data

The 706 European descendant subjects without psychotic disorders included in our study showed a sex ratio close to 1 (53% women) and a mean age of 38.2 (s.d. = 13.4) (% of missing data according to the different countries are available in the online Supplementary Table 3). The scores of subclinical psychosis dimensions and psychosocial stressors scales, and the values of PRS-SZ scores are available in the Table 1 (for non-imputed data, see online Supplementary Table 2).

**Table 1.** Description of the data: socio-demographic, subclinical psychosis, psychosocial stressors and polygenic risk scores variables

	Median (IQR), mean (s.d.) or N (%)
Age	36.00 (22.00), 38.18 (13.35)
Sex	
Women	376 (53.3%)
Men	330 (46.7%)
CAPE dimensions scales	
Positive	4.00 (4.00), 4.51(2.81)
Negative	6.00 (5.00), 6.24 (3.49)
Depressive	4.00 (3.00), 4.39 (1.82)
Psychosocial stressors measures	
Childhood trauma	31.00 (9.75), 33.34 (9.29)
Self-reported discrimination experiences	0.00 (1.00), 0.51 (0.92)
Stressful life events	1.00 (2.00), 1.37 (1.29)
Social capital	0.37 (3.27), 0.37 (2.37)
PRS-SZ	−0.00096 (0.00018), −0.00096 (0.00014)

CAPE, Community Assessment of Psychic Experiences’; IQR, interquartile range; PRS-SZ,  polygenic risk score for schizophrenia; s.d.,  standard-deviation.

### Correlation between genetic vulnerability and environmental factors

Spearman correlation tests did not suggest any evidence of associations between psychosocial stressors levels and the PRS-SZ (Table 2).

**Table 2.** Spearman tests between Z-scores of genetic and environmental factors among subjects with complete data (*N* = 456)

	Childhood trauma	Self-reported discrimination experiences	Stressful life events	Social capital
Polygenic risk score for schizophrenia	*ρ* = 0.063 (*p*-value = 0.18)	*ρ* = −0.047 (*p*-value = 0.32)	*ρ* = 0.083 (*p*-value = 0.08)	*ρ* = 0.032 (*p*-value = 0.48)

Legend: *ρ*: Spearman correlation coefficient.

### Influence of genetic vulnerability and environmental factors on subclinical psychosis dimensions

**Fig. 1.**
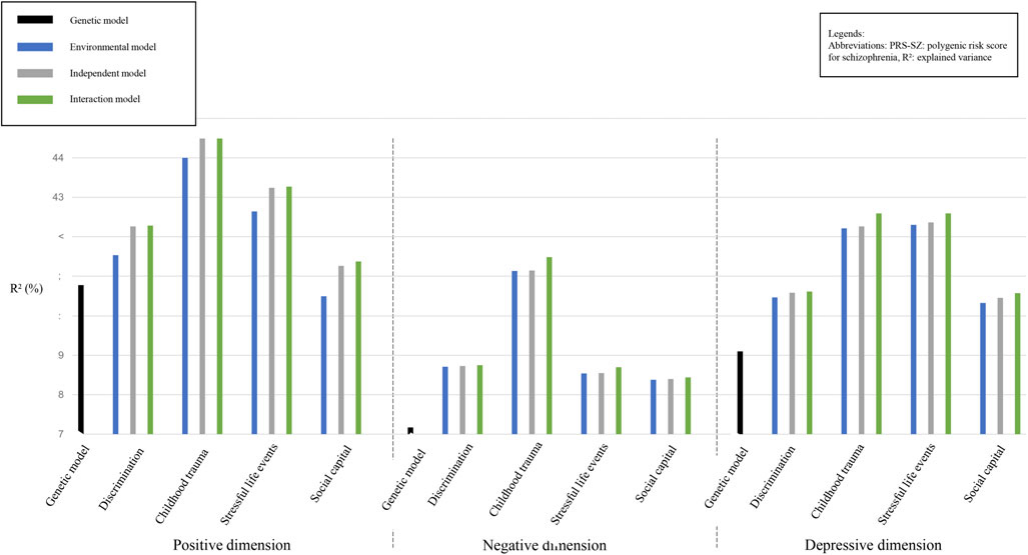
Explained variances of the different models.

For the three subclinical psychosis dimensions that we studied, we first assessed the variance that might be explained by the PRS-SZ (Genetic models, Fig. 1 and Table 3). Only the positive dimension was associated with the PRS-SZ (*β* = 0.086, *p*-value = 0.02, with a *R*^2^ = 7.77%).

**Table 3.** Model comparison of the explained variances of the subclinical psychosis dimensions

			Explained variance by the models (*R*^2^) (%)	PRS-SZ			LRT: comparison of ‘G’ and ‘E + G’ models (*p*-values)	Environmental factor			LRT: comparison of ‘E’ and ‘E + G’ models (*p*-values)	Interaction term between the PRS-SZ and the environmental factor			LRT: comparison of ‘E + G’ and ‘E + G + E × G’ models (*p*-values)
				*β*	s.d.	*p*-value		*β*	s.d.	*p*-value		*β*	s.d.	*p*-value	
Positive dimension	G		7.77	**0**.**086**	**0**.**037**	**0**.**024**	–	–	–	–	–	–	–	–	–
	Self-reported discrimination experiences	E	8.53	–	–	–	–	**0**.**122**	**0**.**037**	**0**.**001**	–	–	–	–	–
		E + G	9.26	**0**.**085**	**0**.**037**	**0**.**026**	**0**.**006**	**0**.**122**	**0**.**037**	**0**.**001**	**0**.**013**	–	–	–	–
		E + G + E × G	9.28	**0**.**085**	**0**.**037**	**0**.**026**	–	**0**.**127**	**0**.**038**	**0**.**001**	–	0.006	0.037	0.776	0.884
	Childhood trauma	E	11.00	–	–	–	–	**0**.**199**	**0**.**036**	**<0**.**001**	–	–	–	–	–
		E + G	11.49	0.069	0.036	0.065	**<0**.**001**	**0**.**193**	**0**.**036**	**<0**.**001**	**0**.**041**	–	–	–	–
		E + G + E × G	11.49	0.069	0.036	0.065	–	**0**.**192**	**0**.**037**	**<0**.**001**	–	0.004	0.036	0.887	0.937
	Stressful life events	E	9.64	–	–	–	–	**0**.**162**	**0**.**036**	**<0**.**001**	–	–	–	–	–
		E + G	10.24	**0**.**076**	**0**.**036**	**0**.**044**	**<0**.**001**	**0**.**157**	**0**.**036**	**<0**.**001**	**0**.**037**	–	–	–	–
		E + G + E × G	10.27	**0**.**076**	**0**.**037**	**0**.**045**	–	**0**.**157**	**0**.**037**	**<0**.**001**	–	−0.007	0.037	0.714	0.652
	Low level of social capital	E	7.50	–	–	–	–	0.069	0.037	0.087	–	–	–	–	–
		E + G	8.26	**0**.**086**	**0**.**037**	**0**.**024**	0.066	0.068	0.037	0.088	**0**.**013**	–	–	–	–
		E + G + E × G	8.37	**0**.**085**	**0**.**037**	**0**.**026**	–	0.066	0.037	0.101	–	−0.029	0.036	0.470	0.650
Negative dimension	G		4.17	0.014	0.038	0.718	–	–	–	–	–	–	–	–	–
	Self-reported discrimination experiences	E	5.71	–	–	–	–	**0**.**125**	**0**.**037**	**0**.**001**	–	–	–	–	–
		E + G	5.73	0.012	0.037	0.748	**<0**.**001**	**0**.**125**	**0**.**037**	**0**.**001**	0.910	–	–	–	–
		E + G + E × G	5.75	0.012	0.037	0.747	–	**0**.**125**	**0**.**037**	**0**.**001**	–	−0.012	0.038	0.740	0.777
	Childhood trauma	E	8.13	–	–	–	–	**0**.**200**	**0**.**037**	**<0**.**001**	–	–	–	–	–
		E + G	8.14	−0.004	0.037	0.854	**<0**.**001**	**0**.**200**	**0**.**037**	**<0**.**001**	0.723	–	–	–	–
		E + G + E × G	8.48	−0.006	0.037	0.838	–	**0**.**213**	**0**.**038**	**<0**.**001**	–	−0.057	0.036	0.125	0.188
	Stressful life events	E	5.54	–	–	–	–	**0**.**117**	**0**.**037**	**0**.**007**	–	–	–	–	–
		E + G	5.55	0.006	0.038	0.830	**<0**.**001**	**0**.**117**	**0**.**037**	**0**.**007**	0.883	–	–	–	–
		E + G + E × G	5.70	0.005	0.038	0.835	–	**0**.**118**	**0**.**037**	**0**.**006**	–	−0.035	0.038	0.386	0.304
	Low level of social capital	E	5.38	–	–	–	–	**0**.**110**	**0**.**037**	**0**.**008**	–	–	–	–	–
		E + G	5.40	0.014	0.037	0.723	**<0**.**001**	**0**.**109**	**0**.**037**	**0**.**009**	0.897	–	–	–	–
		E + G + E × G	5.44	0.014	0.037	0.712	–	**0**.**111**	**0**.**037**	**0**.**008**	–	0.016	0.037	0.660	0.870
Depressive dimension	G		6.10	0.036	0.037	0.350	–	–	–	–	–	–	–	–	–
	Self-reported discrimination experiences	E	7.47	–	–	–	–	**0**.**122**	**0**.**037**	**0**.**001**	–	–	–	–	–
		E + G	7.59	0.034	0.037	0.369	**<0**.**001**	**0**.**122**	**0**.**037**	**0**.**001**	0.373	–	–	–	–
		E + G + E × G	7.62	0.034	0.037	0.369	–	**0**.**123**	**0**.**037**	**0**.**001**	–	−0.017	0.038	0.656	0.489
	Childhood trauma	E	9.21	–	–	–	–	**0**.**180**	**0**.**037**	**<0**.**001**	–	–	–	–	–
		E + G	9.26	0.018	0.037	0.595	**<0**.**001**	**0**.**178**	**0**.**037**	**<0**.**001**	0.611	–	–	–	–
		E + G + E × G	9.59	0.022	0.037	0.566	–	**0**.**165**	**0**.**038**	**<0**.**001**	–	0.056	0.036	0.124	0.058
	Stressful life events	E	9.30	–	–	–	–	**0**.**181**	**0**.**037**	**<0**.**001**	–	–	–	–	–
		E + G	9.36	0.024	0.036	0.525	**<0**.**001**	**0**.**180**	**0**.**037**	**<0**.**001**	0.604	–	–	–	–
		E + G + E × G	9.59	0.022	0.037	0.559	–	**0**.**182**	**0**.**037**	**<0**.**001**	–	−0.047	0.037	0.239	0.325
	Low level of social capital	E	7.33	–	–	–	–	**0**.**115**	**0**.**037**	**0**.**004**	–	–	–	–	–
		E + G	7.46	0.035	0.037	0.354	**0**.**014**	**0**.**115**	**0**.**037**	**0**.**004**	0.363	–	–	–	–
		E + G + E × G	7.58	0.036	0.037	0.338	–	**0**.**118**	**0**.**037**	**0**.**004**	–	0.032	0.037	0.408	0.337

E, Environmental model; E + G, Independent model; E + G + E × G, Interaction model; G, Genetic model; LRT, Likelihood ratio test; PRS-SZ, polygenic risk score for schizophrenia.

The different models were adjusted on age, sex, and the first ten principal components of the ethnicity-based genetic variance.

The significant associations are shown in bold.

We then assessed the variance explained by each of the 4 psychosocial stressors, i.e. discrimination, childhood trauma, stressful events and low social capital (Environmental models). Each psychosocial stressor was associated with the three subclinical psychosis dimensions, except the low level of social capital that was not associated with the positive dimension (Fig. 1 and Table 3). Of note, when associated with subclinical dimensions, the variance explained by Environmental models was always higher than the one explained by Genetic models (except, concerning the positive dimension, for the low level of social capital).

### Combination of genetic and environmental factors

In the Independent models, for the 3 dimensions, the explained variances were better than in the Genetic models, which was confirmed by the LRT (*p*-values < 0001 for almost all models, Fig. 1 and Table 3**,** except for the low level of social capital in the positive dimension).

However, concerning the negative and depressive dimensions, in comparison to Environmental models, the Independent models did not fit better. In other words, adding the PRS-SZ to the Environmental factors did not improve the explained variances of these models. Concerning the positive dimension, the Independent models fitted better than both Genetic and Environment models (LRT: *p*-values between 0.013 and 0.041, Table 3).

In the Interaction models, no significant GxE interaction was observed: adding a GxE interaction term in the Independent models was associated with modest increases of the explained variance, and no interaction term was significantly associated with one of the 3 subclinical psychosis dimension scores. The LRT confirmed that the Interaction models did not fit better than Independent models (Table 3). The analyses presented in Table 3 were repeated in a sample without imputation of the CAPE, without significant change (see online Supplementary Table 3).

## Discussion

In this population-based subjects without psychotic disorders transnational study on the relationships between subclinical psychosis and genetic and environmental (psychosocial stressors) risk factors, the PRS-SZ was associated with the positive dimension but not with the negative and the depressive dimensions. By contrast, the psychosocial stressors were positively associated with the 3 dimensions, except for the low level of social capital, which was not associated with the positive dimension. Moreover, considering the positive dimension, PRS-SZ and psychosocial stressors were independently associated, without GxE interaction, consistently with independent effects of genetic and environmental risk factors.

### A genetic psychotic continuum?

The association between the PRS-SZ and the positive dimension is consistent with the hypothesis of an aetiological psychotic continuum, with subclinical psychosis and psychotic disorders sharing aetiological – genetic and environmental – factors (Linscott and van Os, 2013). This hypothesis could not be verified concerning the other dimensions. A precedent EU-GEI study analysing in controls the relationships between subclinical psychosis and another factor associated with the risk of psychotic disorders, i.e. advanced paternal age, found consistent results: significant association with the positive dimension, but not to negative and depressive dimensions (Schürhoff *et al*., 2020). The aetiological psychotic continuum could concern particularly the positive dimension. Indeed, in comparison to the negative and depressive dimensions, the positive symptoms are the most specific of psychotic disorders (Hanssen *et al*., 2003). Furthermore, in a study analysing the associations between the PRS-SZ and clinical dimensions among antipsychotic-naïve patients with first episode of psychotic disorders (FEP), Santoro *et al*. (2018) found an association with the positive dimension of the positive and negative syndrome scale (PANSS). Moreover, Markota *et al*. (2018) found higher level PRS-SZ in manic-psychosis among patient with bipolar disorder. In future studies, it would be interesting to analyse the relationships between the negative and the depressive dimensions with other PRS (e.g. for depression, or bipolar disorder).

Several studies have found an association between the PRS-SZ and subclinical psychosis, but not all. Indeed, some of these studies did not find any significant associations (Derks *et al*., 2012; Nenadić *et al*., 2020). Methodological differences could be involved, including study population [e.g. some of them were conducted in the paediatric population (Zammit *et al*., 2014; Pries *et al*., 2020*a*)], or the tools used to measure subclinical psychosis [e.g. schizotypy scales do not take account of hallucinations (Seiler *et al*., 2020)]. In a recent study from EU-GEI WP6 (‘*Vulnerability and Severity*’) sample, van Os *et al*. (2020) did not find any associations between PRS-SZ and the 3 dimensions of the CAPE in the controls (without psychotic disorders), although among siblings, a significant association with the negative dimension was found. Among the different studies on association between the PRS-SZ and subclinical psychosis, UK Biobank represents the most closely related to EU-GEI (sample from the general population from United Kingdom), and two of the three studies conducted in UK Biobank found significant associations (Legge *et al*., 2019; García-González *et al*., 2020), contrary to the third, that did not find any significant difference (Alloza *et al*., 2020). Of note, in these 3 UK Biobank studies, the samples were different, especially according to the available data of each subject (e.g. MRI data).

### Association between environmental and genetic factors

Several studies found G-E associations. In a transnational study (Australia, Netherlands and United Kingdom), the PRS-SZ was associated with the population density of the residence (Colodro-Conde *et al*., 2018), i.e. with urbanicity (Vassos *et al*., 2012). These findings were replicated recently in the United Kingdom (Maxwell *et al*., 2021). Of note, this last study considered also other PRS (for depression, bipolar disorder, etc.) and found analogous results. Other studies found similar associations with the cannabis use (Gage *et al*., 2017; Pasman *et al*., 2018). These studies suggest that the association between these environmental factors and the risk of psychotic disorders could partially be explained by the same genetic factors (Pingault *et al*., 2018). This hypothesis particularly concerns childhood trauma, that has often be supposed to be associated with vulnerabilities to psychiatric disorders (Etain *et al*., 2008; Varese *et al*., 2012; Baudin *et al*., 2017). Sharing the same genetic risk factors could explain the association between childhood trauma and psychiatric disorders. However, in our study, we did not find any G-E association neither with childhood trauma nor with the other psychosocial stressors.

### Gene x environment (psychosocial stressors) interactions

Our study did not show any statistically significant interaction between the psychosocial stressors and the PRS-SZ, but independent effects concerning the positive dimension. Trotta *et al*. (2016) found similar results: the PRS-SZ and childhood trauma history predicted both psychosis status, without interaction between these factors. To our knowledge, two other studies have looked for such interactions, that found a significant GxE interaction between the PRS-SZ and childhood trauma (Pries *et al*., 2020*b*; Schick *et al*., 2022). Another study using the PRS-SZ and conducted in adults looked for interaction with other environmental factors, i.e. smoking status, without finding any GxE interaction (García-González *et al*., 2020).

One hypothesis to explain the negative results of this GxE interaction study is that the PRS-SZ is not the appropriate tool for the study of GxE interaction in psychosis (Assary *et al*., 2018). Indeed, this statistical tool summarises essentially monogenic factors with small effects sizes; and GxE interaction could only involve monogenic factors (Caspi *et al*., 2005; Stefanis *et al*., 2007; Alemany *et al*., 2011). However, other studies used PRS and found GxE interaction, for instance between childhood trauma and the PRS for depression in the risk of major depressive disorder (Peyrot *et al*., 2014), or between this PRS and stressful life events in the level of depressive symptoms (Domingue *et al*., 2017), as well as studies on non-psychiatric diseases, e.g. for breast cancer (Meisner *et al*., 2019; Shi *et al*., 2020). The problem could concern specifically PRS-SZ, with (i) an insufficient sample of subjects included in the genome-wide association studies (GWAS) used to calculate it, which is a major issue concerning PRSs (Plomin and von Stumm, 2018), and (ii) the fact that the PRS-SZ performed better among European descendants (which has prevented the inclusion of subjects from ethnic minorities) (Vassos *et al*., 2017). Moreover, the PRS does not take copy number variant (CNVs) or epigenetic factors in account, and they are associated with the risk of schizophrenia (and with childhood trauma concerning epigenetic factors) (St Clair, 2009; Shorter and Miller, 2015; Parade *et al*., 2021). Another hypothesis states that the genes that increase the sensibility to environmental stressors could be different from the genes that increase the risk of schizophrenia (displayed in the GWAS). Furthermore, GxE interactions could also concern other environmental factors (urbanicity, advanced paternal age, migration, etc.). Finally, the study GxE interactions using exposome scores (Pries *et al*., 2020*a*), that takes account of several environmental exposures (including psychosocial stressors), could be instructive.

## Limitations

Some limitations should be acknowledged. First, due to the cross-sectional nature of EU-GEI study, the assessment of both subclinical psychosis and psychosocial stressors was retrospective, thus susceptible to be biased (e.g. recall bias) and influenced by clinical variables as depressive or positive symptoms (MacDonald *et al*., 2015). These potential biases, especially concerning psychosocial stressors assessment (particularly the low level of discrimination experience), have been discussed previously (Pignon *et al*., 2021). Moreover, regarding the sample size, that could be considered as insufficient to enhance an GxE interaction, Pries *et al*. (2020*a*) found an interaction between childhood adversity and PRS-SZ concerning subclinical psychosis (with an ecological momentary assessment) with a lower sample (*n* = 593). The absence of subjects from ethnic minorities, is a major limitation (Tortelli *et al*., 2018). Indeed, these minorities are exposed to higher levels of psychosocial stress (Hatch *et al*., 2016). Contrary to the CAPE (Pignon *et al*., 2019*b*), concerning the assessment of these psychosocial stressors, the cross-national invariance of the different tools that were used (CTQ, Williams' major experiences of discrimination scale, LTE, SEAT) has not been studies. Moreover, as the sampling was not fully at random, we cannot assume that our sample was representative of the general population.

## Conclusion

This general population-based study revealed an association between PRS-SZ and the subclinical positive dimension of psychosis, as well as independent effects of the PRS-SZ and of the psychosocial stressors (childhood trauma, stressful life events, self-reported discrimination experiences) on the positive dimension, contrary to the negative and depressive dimensions. Moreover, concerning the 3 dimensions, this study did not evidence any GxE interaction, or any G-E association.

## Data Availability

Data is confidential and not available.

## Supplementary material

For supplementary material accompanying this paper visit http://doi.org/10.1017/S2045796022000464. click here to view supplementary material
