# Supplementary material for: Genetic and psychosocial stressors have independent effects on the level of subclinical psychosis: findings from the multinational EU-GEI study
Source: Epidemiol Psychiatr Sci. 2022 Sep 27;31:e68. doi: 10.1017/S2045796022000464 (PMC9533114; doi:10.1017/S2045796022000464)
Supplement: Supplementary file 1 [file epssup.zip › S2045796022000464sup001.docx]

| **Supplementary Table 2: Description of the unimputed data: socio-demographic, subclinical psychosis, psychosocial stressors and polygenic risk score variables.** | |
| --- | --- |
|  | Median (IQR), mean (sd) |
| *CAPE dimensions scales* | |
| Positive | 4.00 (4.00), 4.49 (2.76) |
| Negative | 6.00 (6.00), 6.13 (3.50) |
| Depressive | 4.00 (2.25), 4.31 (1.78) |
| *Psychosocial stressors measures* | |
| Childhood trauma | 31.00 (11.00), 33.43 (9.66) |
| Self-reported discrimination experiences | 0.00 (1.00), 0.43 (0.82) |
| Stressful life events | 1.00 (2.00), 1.36 (1.30) |
| Social capital | 0.58 (3.25), 0.48 (2.42) |
| PRS-SZ | -0.00095 (0.00017), -0.00097 (0.00014) |
| Abbreviations: CAPE = Community Assessment of Psychic Experiences, IQR = interquartile range, PRS-SZ = polygenic risk score for schizophrenia, sd = standard-deviation. | |
