# Supplementary material for: Genetic and psychosocial stressors have independent effects on the level of subclinical psychosis: findings from the multinational EU-GEI study
Source: Epidemiol Psychiatr Sci. 2022 Sep 27;31:e68. doi: 10.1017/S2045796022000464 (PMC9533114; doi:10.1017/S2045796022000464)
Supplement: Supplementary file 1 [file epssup.zip › S2045796022000464sup002.docx]

| **Supplementary Table 3: Model comparison of the explained variances of the subclinical psychosis dimensions** | | | | | | | | | | | | | | | | |
| --- | --- | --- | --- | --- | --- | --- | --- | --- | --- | --- | --- | --- | --- | --- | --- | --- |
|  | Number of non-available data of the CAPE |  | | Explained variance by the models (R²) | PRS-SZ | | | LRT: comparison of “G” and  “E + G” models  (p-values) | Environmental factor | | | LRT: comparison of “E” and  “E + G” models  (p-values) | Interaction term between the PRS-SZ and the environmental factor | | | LRT: comparison of “E + G” and  “E + G + E*G” models  (p-values) |
|  |  |  |  |  | ß | sd | p-value |  | ß | sd | p-value |  | ß | sd | p-value |  |
| Positive dimension | 42 | G | | 8.40 % | **0.094** | **0.038** | **0.015** | - | - | - | - | - | - | - | - | - |
|  |  | Self-reported discrimination experiences | E | 9.19 % | - | - | - | - | **0.129** | **0.038** | **<0.001** | - | - | - | - | - |
|  |  |  | E + G | 10.00 % | **0.091** | **0.038** | **0.017** | **< 0.001** | **0.127** | **0.038** | **0.001** | **0.013** | - | - | - | - |
|  |  |  | E + G + E*G | 10.02 % | **0.091** | **0.038** | **0.017** | **-** | **0.127** | **0.038** | **0.001** | - | 0.008 | 0.039 | 0.833 | 0.809 |
|  |  | Childhood trauma | E | 11.62 % | - | - | - | **-** | **0.199** | **0.037** | **< 0.001** | - | - | - | - | - |
|  |  |  | E + G | 12.17 % | 0.076 | 0.038 | 0.045 | **< 0.001** | **0.192** | **0.037** | **< 0.001** | **0.046** | - | - | - | - |
|  |  |  | E + G + E*G | 12.18 % | 0.076 | 0.038 | 0.045 | **-** | **0.191** | **0.038** | **< 0.001** | - | 0.004 | 0.036 | 0.918 | 0.945 |
|  |  | Stressful life events | E | 9.47 % | - | - | - | **-** | **0.138** | **0.038** | **< 0.001** | - | - | - | - | - |
|  |  |  | E + G | 10.17 % | **0.085** | **0.038** | **0.025** | **< 0.001** | **0.133** | **0.038** | **0.001** | **0.020** | - | - | - | - |
|  |  |  | E + G + E*G | 10.19 % | **0.085** | **0.038** | **0.026** | **-** | **0.133** | **0.038** | **0.001** | **-** | -0.003 | 0.039 | 0.806 | 0.862 |
|  |  | Low level of social capital | E | 8.04 % | - | - | - | **-** | 0.070 | 0.038 | 0.077 | **-** | - | - | - | - |
|  |  |  | E + G | 8.90 % | **0.094** | **0.038** | **0.015** | 0.077 | 0.070 | 0.038 | 0.075 | **0.013** | - | - | - | - |
|  |  |  | E + G + E*G | 9.00 % | **0.093** | **0.038** | **0.016** | **-** | 0.068 | 0.038 | 0.085 | - | -0.030 | 0.0384 | 0.460 | 0.759 |
| Negative dimension | 34 | G | | 4.58 % | 0.016 | 0.039 | 0.688 | **-** | - | - | - | - | - | - | - | - |
|  |  | Self-reported discrimination experiences | E | 6.63 % | - | - | - | **-** | **0.136** | **0.039** | **< 0.001** | - | - | - | - | - |
|  |  |  | E + G | 6.33 % | 0.017 | 0.038 | 0.661 | **< 0.001** | **0.136** | **0.039** | **< 0.001** | 0.636 | - | - | - | - |
|  |  |  | E + G + E*G | 6.34 % | 0.017 | 0.039 | 0.663 | **-** | **0.136** | **0.039** | **< 0.001** | - | -0.003 | 0.041 | 0.900 | 0.895 |
|  |  | Childhood trauma | E | 8.87 % | - | - | - | **-** | **0.207** | **0.037** | **< 0.001** | - | - | - | - | - |
|  |  |  | E + G | 8.87 % | - 0.002 | 0.038 | 0.956 | **< 0.001** | **0.206** | **0.037** | **< 0.001** | 0.918 | - | - | - | - |
|  |  |  | E + G + E*G | 9.35 % | - 0.002 | 0.038 | 0.911 | - | **0.222** | **0.038** | **< 0.001** | - | -0.068 | 0.037 | 0.067 | 0.037 |
|  |  | Stressful life events | E | 5.86 % | - | - | - | - | **0.114** | **0.038** | **0.006** | - | - | - | - | - |
|  |  |  | E + G | 5.87 % | 0.008 | 0.038 | 0.828 | **< 0.001** | **0.113** | **0.039** | **0.007** | 0.782 | - | - | - | - |
|  |  |  | E + G + E*G | 6.03 % | 0.006 | 0.038 | 0.870 | **-** | **0.115** | **0.038** | **0.006** | - | -0.040 | 0.040 | 0.332 | 0.477 |
|  |  | Low level of social capital | E | 5.48 % | - | - | - | **-** | **0.097** | **0.039** | **0.016** | - | - | - | - | - |
|  |  |  | E + G | 5.51 % | 0.014 | 0.039 | 0.703 | **0.014** | **0.096** | **0.039** | **0.017** | 0.696 | - | - | - | - |
|  |  |  | E + G + E*G | 5.53 % | 0.014 | 0.039 | 0.701 | **-** | **0.097** | **0.039** | **0.017** | - | 0.004 | 0.039 | 0.746 | 0.994 |
| Depressive dimension | 24 | G | | 6.61 % | 0.041 | 0.038 | 0.280 | **-** | - | - | - | - | - | - | - | - |
|  |  | Self-reported discrimination experiences | E | 7.94 % | - | - | - | **-** | **0.124** | **0.038** | **0.001** | - | - | - | - | - |
|  |  |  | E + G | 8.10 % | 0.040 | 0.038 | 0.287 | **< 0.001** | **0.124** | **0.038** | **0.001** | 0.270 | - | - | - | - |
|  |  |  | E + G + E*G | 8.12 % | 0.040 | 0.038 | 0.540 | **-** | **0.124** | **0.038** | **0.001** | - | -0.013 | 0.038 | 0.745 | 0.716 |
|  |  | Childhood trauma | E | 9.67 % | - | - | - | **-** | **0.181** | **0.037** | **< 0.001** | - | - | - | - | - |
|  |  |  | E + G | 9.72 % | 0.023 | 0.037 | 0.537 | **< 0.001** | **0.179** | **0.037** | **< 0.001** | 0.563 | - | - | - | - |
|  |  |  | E + G + E*G | 10.02 % | 0.025 | 0.037 | 0.515 | **-** | **0.164** | **0.039** | **< 0.001** | - | 0.054 | 0.037 | 0.144 | 0.114 |
|  |  | Stressful life events | E | 8.84 % | - | - | - | **-** | **0.154** | **0.037** | **< 0.001** | - | - | - | - | - |
|  |  |  | E + G | 8.93 % | 0.031 | 0.038 | 0.405 | **< 0.001** | **0.152** | **0.037** | **< 0.001** | 0.350 | - | - | - | - |
|  |  |  | E + G + E*G | 9.18 % | 0.030 | 0.038 | 0.430 | **-** | **0.154** | **0.037** | **< 0.001** | - | -0.048 | 0.038 | 0.243 | 0.191 |
|  |  | Low level of social capital | E | 7.68 % | - | - | - | **-** | **0.111** | **0.038** | **0.006** | - | - | - | - | - |
|  |  |  | E + G | 7.86 % | 0.041 | 0.037 | 0.273 | **< 0.001** | **0.111** | **0.038** | **0.007** | 0.272 | - | - | - | - |
|  |  |  | E + G + E*G | 7.95 % | 0.042 | 0.038 | 0.263 | - | **0.112** | **0.038** | **0.006** | - | 0.028 | 0.038 | 0.477 | 0.165 |
| The different models were adjusted on age, sex, and the first ten principal components of the ethnicity-based genetic variance.  The significant associations are shown in bold.  Abbreviations: E: Environmental model, E + G: Independent model, E + G + E*G: Interaction model, G: Genetic model, LRT: Likelihood ratio test, PRS-SZ: polygenic risk score for schizophrenia. | | | | | | | | | | | | | | | | |
