# Supplementary material for: Genetic and psychosocial stressors have independent effects on the level of subclinical psychosis: findings from the multinational EU-GEI study
Source: Epidemiol Psychiatr Sci. 2022 Sep 27;31:e68. doi: 10.1017/S2045796022000464 (PMC9533114; doi:10.1017/S2045796022000464)
Supplement: Supplementary file 1 [file epssup.zip › S2045796022000464sup003.docx]

| **Supplementary Table 1: Effectives and rates of missing data according to the different countries** | | | | | | |
| --- | --- | --- | --- | --- | --- | --- |
|  | Brazil  N = 62 | France  N = 71 | Holland  N = 120 | Italy  N = 147 | Spain  N = 129 | United-Kingdom  N = 177 |
| *CAPE dimensions scales* | | | | | | |
| Positive | 0 (0%) | 13 (18.31%) | 8 (6.67%) | 6 (4.08%) | 13 (10.08%) | 2 (1.13%) |
| Negative | 0 (0%) | 14 (19.72%) | 7 (5.83%) | 5 (3.40%) | 4 (3.10%) | 4 (2.26%) |
| Depressive | 0 (0%) | 4 (5.63%) | 8 (6.67%) | 4 (2.72%) | 6 (4.65%) | 2 (1.13%) |
| *Psychosocial stressors measures* | | | | | | |
| Childhood trauma | 0 (0%) | 0 (0%) | 0 (0%) | 2 (1.36%) | 1 (0.78%) | 1 (0.56%) |
| Self-reported discrimination experiences | 0 (0%) | 0 (0%) | 2 (1.67%) | 10 (6.80%) | 7 (5.43%) | 5 (2.82%) |
| Stressful life events | 3 (4.83%) | 4 (5.63%) | 5 (4.17%) | 94 (63.95%) | 38 (29.46%) | 2 (1.13%) |
| Social capital | 3 (4.83%) | 17 (23.94%) | 10 (8.33%) | 38 (25.85%) | 19 (14.73%) | 3 (1.69%) |
| Abbreviations: CAPE = Community Assessment of Psychic Experiences | | | | | | |
